# Comparative Efficacy of State-of-the-Art and New Biological Stump Treatments in Forests Infested by the Native and the Alien Invasive *Heterobasidion* Species Present in Europe

**DOI:** 10.3390/pathogens10101272

**Published:** 2021-10-02

**Authors:** Martina Pellicciaro, Guglielmo Lione, Silvia Ongaro, Paolo Gonthier

**Affiliations:** Department of Agricultural, Forest and Food Sciences (DISAFA), University of Turin, 10095 Grugliasco, Italy; martina.pellicciaro@unito.it (M.P.); guglielmo.lione@unito.it (G.L.); silvia.ongaro@unito.it (S.O.)

**Keywords:** biological control, cell-free filtrate, forest pathogens, *Phlebiopsis gigantea*, Proradix®, *Pseudomonas protegens*, Rotstop®, urea

## Abstract

The *Heterobasidion annosum* species complex includes major fungal pathogens of conifers worldwide. State-of-the-art preventative stump treatments with urea or with commercial formulations of the fungal biological control agent *Phlebiopsis gigantea* (i.e., Rotstop^®^) may become no longer available or are not approved for use in many areas of Europe infested by the three native *Heterobasidion* species and by the North American invasive *H. irregulare*, making the development of new treatments timely. The efficacy of Proradix^®^ (based on *Pseudomonas protegens* strain DSMZ 13134), the cell-free filtrate (CFF) of the same bacterium, a strain of *P. gigantea* (MUT 6212) collected in the invasion area of *H. irregulare* in Italy, Rotstop^®^, and urea was comparatively investigated on a total of 542 stumps of *Abies alba*, *Picea abies, Pinus pinea*, and *P. sylvestris* in forest stands infested by the host-associated *Heterobasidion* species. Additionally, 139 logs of *P. pinea* were also treated. Results support the good performances of Rotstop^®^, and especially of urea against the native *Heterobasidion* species on stumps of their preferential hosts and, for the first time, towards the invasive North American *H. irregulare* on stumps of *P. pinea*. In some experiments, the effectiveness of Proradix^®^ and of the strain of *P. gigantea* was weak, whereas the CFF of *P. protegens* strain DSMZ 13134 performed as a valid alternative to urea and Rotstop^®^. The mechanism of action of this treatment hinges on antibiosis; therefore, further improvements could be possible by identifying the active molecules and/or by optimizing their production. Generally, the performance of the tested treatments is not correlated with the stump size.

## 1. Introduction

*Heterobasidion annosum* (Fr.) Bref. *sensu lato* (*s.l.*) is a complex of species comprising fungal plant pathogens causing root rots, butt rots and wood decay in coniferous forests of the Northern Hemisphere [1,2]. Annual economic losses caused by *H. annosum s.l.* in Europe were estimated at EUR 790 million [1] and occur in association with the decrease in wood production and the reduction in wood quality, which are reported as directly correlated to the incidence of the pathogens [3]. In Europe, *H. annosum s.l.* includes the native species *H. abietinum* Niemelä & Korhonen, *H. annosum sensu strictu (s.s.)* and *H. parviporum* Niemelä & Korhonen, mainly associated with *Abies alba* Mill., *Pinus* spp., and *Picea abies* (L.) Karst., respectively [2]. In addition, Italian stone pine (*Pinus pinea* L.) is located along the western coastline of central Italy is currently challenged by the invasive North American species *H. irregulare* Garbel. & Otrosina [4,5]. The risk posed by *H. irregulare* to European forestry is high [6]; therefore, the pathogen is recommended for regulation under the European and Mediterranean Plant Protection Organization (EPPO) A2 list.

*Heterobasidion* spp. infect their hosts through basidiospores or mycelium. Primary infection occurs when airborne basidiospores released from fruiting bodies land on the surface of freshly cut stumps and germinate, hence producing mycelia able to colonize the stump tissues. Once the root system of the stump is colonized, the mycelium of *Heterobasidion* spp. spreads to the neighboring heathy trees by means of root contacts and grafts [2]. This second method of spreading is acknowledged as secondary infection. Hence, stumps created during thinnings and cuttings play a crucial role as starters for new infection foci. 

Silvicultural, chemical and biological strategies have been designed and tested to control *Heterobasidion* spp. infections and spreading [2]. Most of the silvicultural methods are based on providing spacing among trees in new plantations, on planning thinnings and cuttings during periods of low sporulation of the pathogens, and on the mechanical removal of stumps of diseased trees, including their root systems [7,8,9,10,11].

Although effective in reducing the risk of new infections, such strategies imply remarkable technical challenges and high costs for their execution; hence, they may be either unfeasible, or scarcely practicable [12,13,14]. Conversely, chemical or biological treatments applied on freshly cut stumps act as preventative measures targeting primary infection and are recognized as more sustainable than most of the other strategies [2,13].

A large body of literature has been published on the efficacy of urea [10,15,16,17]. The mechanism of action of urea has been reported as indirect. The urea solution raises the pH of the stump up to a level that inhibits basidiospore survival and germination [18]. Urea is the only chemical treatment used in practical forestry in Europe. However, the current European regulations might pose some constraints for the future use of urea, unless an extension of its authorization as a pesticide is issued after the deadline of August 2022 [19].

In the attempt to reduce the use of chemicals in forestry, several studies have focused on the selection of microorganisms that may act as biocontrol agents and inhibit *Heterobasidion* spp. The most successful results were obtained in the field with *Phlebiopsis gigantea* (Fr.) Jülich, a wood decay basidiomycete outcompeting *Heterobasidion* spp. thanks to its rapid colonization of the stump surface [12,16,17,20,21,22,23,24,25,26,27,28].

*Phlebiopsis gigantea* strains patented as Rotstop^®^ are widely used in practical forestry both in Europe and North America [13,29], although neither such treatment, nor other products based on *P. gigantea*, are registered for use in southern European countries. Most of the results currently available on the efficacy of *P. gigantea* originate from experiments conducted against *H. parviporum* on *P. abies*, although information about the performance of *P. gigantea* against other *Heterobasidion* spp. on stumps of other tree species, i.e., *A. alba, P. pinea* and *P. sylvestris*, are still scarce. Moreover, few studies dealt with the comparative assessment of the efficacy of *P. gigantea* and urea on stumps [16,24,28,30].

As a result of preliminary studies, the bacterial biocontrol agent *Pseudomonas protegens* (strain DSMZ 13134), patented as Proradix^®^, proved its efficacy against *Heterobasidion* spp. [31,32,33]. The production of antibiotics and diffusible antifungal compounds allows *P. protegens* (strain DSMZ 13134) to inhibit the growth of *Heterobasidion* spp., as suggested by the outcomes of trials carried out by using the cell-free filtrate (CFF) of *P. protegens* (strain DSMZ 13134) [33]. Although promising, treatments based on *P. protegens* (strain DSMZ 13134) still need to be tested against all *Heterobasidion* species on stumps of their preferential hosts. In fact, so far, available data about *P. protegens* are limited to a small-scale pilot study on stumps of *P. abies*.Stump diameter influences the colonization process of *Heterobasidion* spp. [34,35]. It has been suggested that large areas of heartwood might create more opportunities for *H. parviporum* infection in *P. abies* [11]. However, in several tree species, sapwood is much more extensively infected by *Heterobasidion* spp. than heartwood [36], which is consistent with the observation that the fungus can establish even in small (<2 cm) stumps [37]. Although the effects of stump size on primary infections by *Heterobasidion* spp. have been investigated, the relationship between the treatment efficacy and the stump size has received little attention despite its potential importance in practical forestry [30].Comparative studies contrasting the effectiveness of chemical and biological treatments on freshly cut stumps are advocated to design effective and sustainable control measures against *Heterobasidion* spp. on different host species present in Europe [28], taking into account that the level of efficacy of treatments may vary depending on climate and on local environmental conditions [38,39]. This issue is of the utmost importance not only in relation to the alien species *H. irregulare* threatening *P. pinea* across its invasion area in central Italy, but also for southern European countries, where treatments against *Heterobasidion* spp. are either not registered, or will be soon revoked. The aims of this study were to compare the efficacy of Proradix^®^, the CFF of *P. protegens* strain DSMZ 13134, the strain of *P. gigantea* MUT 6212 collected on *P. pinea* in the *H. irregulare* invasion area in central Italy and screened for its biocontrol potential [40], the biological control agent Rotstop^®^, and the chemical control agent urea as stump treatments in forests of *A. alba*, *P. abies, P. pinea,* and *P. sylvestris* each infested by their own host-associated *Heterobasidion* species (i.e., *H. abietinum*, *H. parviporum, H. irregulare, H. annosum s.s.*, respectively). Our experiments were carried out in two of the main ecoregions where the above cited hosts of *Heterobasidion* spp. grow, namely, the Alps (for *A. alba*, *P. abies*, and *P. sylvestris*) and the Mediterranean area (for *P. pinea*) [41,42]. To further investigate the efficacy of treatments against *H. irregulare*, an experiment was also set up in the field using *P. pinea* logs. An additional and final goal was to explore the effect of stump size on treatment efficacy.

## 2. Results

### 2.1. Comparative Efficacy of Treatments in Stumps of Abies alba, Picea abies, Pinus pinea and P. sylvestris 

**Figure 1:**
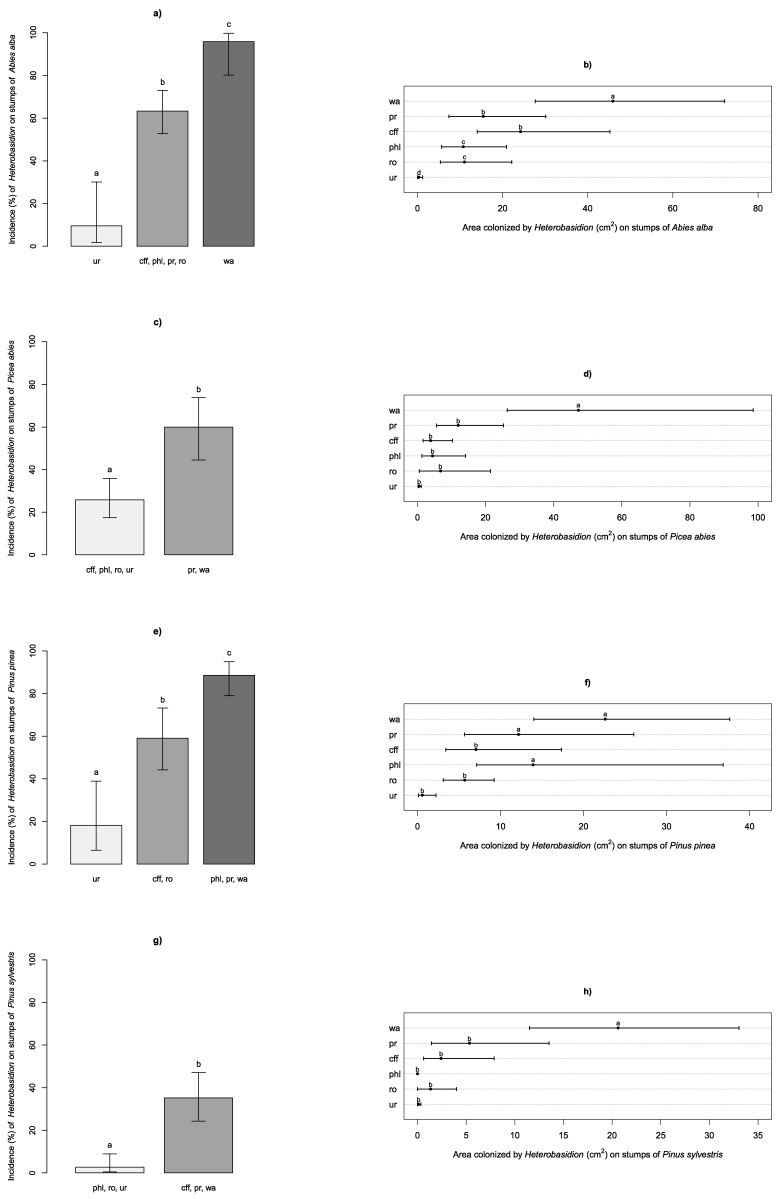
Barplots of the incidence (%—left panels) and dotcharts of the areas colonized by *Heterobasidion* spp. (cm^2^—right panels) on treated stumps of *Abies alba* (panels (**a**,**b**)), *Picea abies* (panels (**c**,**d**)), *Pinus pinea* (panels (**e**,**f**)), *P. sylvestris* (panels (**g**,**h**)). Treatment abbreviations: wa—water; pr—Proradix®; cff—cell-free filtrate; phl—*Phlebiopsis gigantea* MUT 6212; ro—Rotstop®; and ur—urea. For details, refer to the text.

Stumps of *A. alba* treated and sampled were as follows: 24 control stumps sprayed with sterile water, 22 stumps treated with Proradix^®^, 23 with CFF, 23 with *P. gigantea* MUT 6212, 22 with Rotstop^®^ and 21 with urea. Isolates obtained from control stumps of *A. alba* were all typed as *H. abietinum.* Incidence and areas colonized by *Heterobasidion* spp. on wood discs sampled from stumps are shown in Figure 1. In the Figure 1 barplots, each bar refers to a cluster of treatments and shows the related overall incidence of *Heterobasidion* spp. Treatments included within the same cluster are not associated with significant differences (*p* > 0.05), whereas treatments belonging to different clusters did result in significant differences (*p* < 0.05) of the incidence of *Heterobasidion* spp. Abbreviations listed under the bars indicate the treatments included within the corresponding cluster. In the dotcharts of the same figure, point marks show the average areas colonized by *Heterobasidion* spp. for each treatment. In both barplots and dotcharts, different letters indicate significant differences of the associated values (*p* < 0.05), and error bars refer to the 95% confidence intervals. The incidence of the pathogen was significantly (*p* < 0.05) higher in *A. alba* control stumps (95.8%) than in stumps treated with biological products (63.3%), that clustered together and separately from stumps treated with urea (9.5%) (Figure 1a). All treatments resulted in a significant reduction in area colonized by the pathogen compared to controls (Figure 1b). Urea provided the best performance among treatments (0.3% of area colonized by the pathogen), whereas stumps treated with *P. gigantea* MUT 6212 and Rotstop® resulted in a less severe infection of stumps (10.7% and 11.0%, respectively) compared to Proradix^®^ and CFF (15.4% and 24.2%, respectively). 

Stumps of *P. abies* treated and sampled were as follows: 21 control stumps sprayed with sterile water, 20 stumps treated with Proradix^®^, 21 with CFF, 21 with *P. gigantea* MUT 6212, 21 with Rotstop^®^ and 21 with urea. Isolates obtained from control stumps of *P. abies* were all typed as *H. parviporum.* The incidence of the pathogen was significantly higher in control stumps and in stumps treated with Proradix^®^ (60.0%) than in the other treatments (25.8%) (Figure 1c). In *P. abies* stumps, all treatments resulted in areas colonized by the pathogen significantly smaller than in controls (Figure 1d). 

Stumps of *P. pinea* treated and sampled were as follows: 25 control stumps sprayed with sterile water, 23 stumps treated with Proradix^®^, 22 with CFF, 22 with *P. gigantea* MUT 6212, 22 with Rotstop^®^ and 22 with urea. Isolates obtained from control stumps of *P. pinea* were all typed as *H. irregulare.* In *P. pinea* stumps, the incidence of the pathogen was significantly higher in control stumps and in stumps treated with Proradix^®^ and *P. gigantea* MUT 6212 (88.6%) than in stumps treated with CFF and Rotstop® (59.1%) (Figure 1e). Urea performed significantly better than the other treatments (18.2% incidence of the pathogen). All treatments reduced the area colonized by the pathogen compared to control stumps (Figure 1f). However, such a reduction was significant only for CFF, Rotstop^®^ and urea (7.1%, 5.7%, and 0.6%, respectively) (Figure 1f).

Stumps of *P. sylvestris* treated and sampled were as follows: 24 control stumps sprayed with sterile water, 23 stumps treated with Proradix^®^, 24 with CFF, 25 with *P. gigantea* MUT 6212, 24 with Rotstop^®^ and 26 with urea. Isolates obtained from control stumps of *P. sylvestris* were all typed as *H. annosum s.s.* The incidence of the pathogen was significantly higher for control stumps, stumps treated with Proradix® and CFF (35.2%) than for stumps treated with *P. gigantea* MUT 6212, Rotstop® and urea, which showed an incidence of 2.6% (Figure 1g). The area colonized by the pathogen in treated stumps was significantly lower than that in control stumps (20.6%) for all treatments (Figure 1h). Treatment with *P. gigantea* MUT 6212 and with urea resulted in 0% and 0.1% areas colonized by the pathogen, respectively. Stumps treated with Proradix®, CFF and Rotstop^®^ resulted in 5.3%, 2.4% and 1.3% areas colonized by the pathogen, respectively. 

### 2.2. Efficacy of Treatments on Logs of Pinus pinea

**Figure 2:**
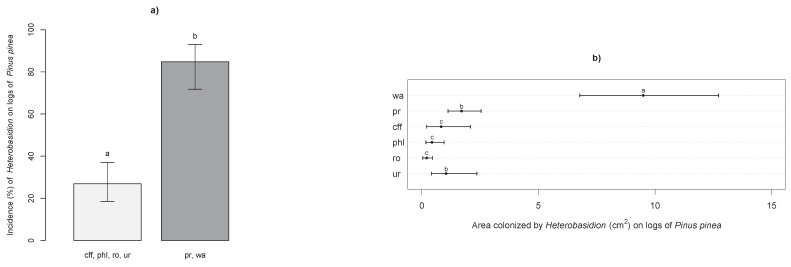
Barplots of the incidence (%—panel (**a**)) and dotcharts of the areas colonized by *Heterobasidion* spp. (cm^2^—panel (**b**)) on treated logs of *Pinus pinea*. Treatment abbreviations: wa—water; pr—Proradix®; cff—cell-free filtrate; phl—*Phlebiopsis gigantea* MUT 6212; ro—Rotstop®; and ur—urea. For details, refer to the text.

Logs of *P. pinea* treated and sampled were as follows: 22 control logs sprayed with sterile water, 24 logs treated with Proradix^®^, 24 with CFF, 23 with *P. gigantea* MUT 6212, 24 with Rotstop^®^ and 22 with urea. Isolates obtained from control logs of *P. pinea* were all typed as *H. irregulare.* Incidence and areas colonized by the pathogen on wood discs gathered from logs are shown in Figure 2. Barplots and dotcharts of Figure 2 were built as shown in Figure 1. The incidence of the pathogen in control logs and in logs treated with Proradix^®^ was significantly higher (84.7%) than that in logs treated with the other treatments (26.8%) (Figure 2a). Significant reductions in the area colonized by the pathogen compared to control logs (9.5% of area colonized) were observed for all treatments (Figure 2b). In terms of the area colonized by the pathogen, CFF, *P. gigantea* MUT 6212 and Rotstop^®^ performed significantly better than Proradix^®^ and urea (0.8%, 0.4 and 0.2% vs. 1.7% and 1.0% of area colonized, respectively). 

### 2.3. Correlation between Stump Size and Efficacy of Treatments 

The correlations between stump size and the area colonized by *Heterobasidion* spp. on wood discs sampled from stumps are shown in Table 1. For *P. abies, P. pinea,* and *P. sylvestris*, there were no significant correlations, either in control or in treated stumps. 

For *A. alba*, a significant correlation between stump size and the area colonized by the pathogen was observed in control stumps (R = 0.474; *p =* 0.019) and in stumps treated with Proradix^®^ (R = 0.447; *p* = 0.037). The other treatments did not display significant correlations between the two variables.

## 3. Discussion

In this study, four biological stump treatments (Proradix^®^, CFF, *P. gigantea* MUT 6212 and Rotstop^®^) and one chemical treatment (urea) were tested against naturally occurring primary infections operated by all *Heterobasidion* species present in Europe, including the invasive *H. irregulare*, on their preferential hosts. Since the pioneering work of Rishbeth [43,44,45,46], who first elucidated the infection biology of the fungal pathogen and the potentiality of stump treatments for disease control, several studies have reported the effectiveness of stump treatments against primary infections of *Heterobasidion* spp. (see [10,20,22,24,28,30,47] and literature therein), but only a few of them compared the efficacy of biological and chemical control agents, namely, urea, in the same experiments [15,16,48]. In addition, biological control with bacteria, although promising and effective in vitro against forest pathogens [33,49,50,51], have never been tested in the field thus far, with the exception of a pilot study conducted on a few *P. abies* stumps [31,32]. Overall, with the exception of Proradix^®^ and *P. gigantea* MUT 6212 on *P. pinea* stumps, all biological and chemical treatments tested were effective in significantly reducing the area colonized by *Heterobasidion* spp. on stumps of the tested tree species compared to controls. Furthermore, the efficacy of treatments was not correlated with stump size, with Proradix^®^ on *A. alba* stumps being the only exception.

The experiments were carried out in periods at high risk for primary infections of *Heterobasidion* spp. in the Alps (i.e., in summer and autumn) and in coastal regions of central Italy (i.e., in winter), based on previously published information on the availability of airborne inoculum or on the frequency of stump infections [9,10,52]. This allowed relying on natural airborne infections for the experiments, without the need for artificial inoculations of the pathogens. Indeed, results of the experiments suggest that the risk of stump infection by *H. annosum s.l.* may be relevant where treatments are not ordinarily carried out on freshly cut stumps. It was previously suggested that stump treatments are economically profitable when stump infection frequency in untreated stumps reaches or exceeds the threshold of 20% to 30% [53,54]. In the present study, the incidence of the pathogen in control stumps ranged from 50% to 95.8% depending on tree species, thus recommending stump treatments in such situations.

Study sites were pre-selected based on the availability of airborne inoculum of the *Heterobasidion* species commonly associated with the most abundant tree species in the forest stands. Indeed, all isolates obtained from control stumps in each stand were identified as belonging the *Heterobasidion* species predicted. Therefore, results of our treatment experiments on *A. alba*, *P. abies*, *P. pinea* and *P. sylvestris* stumps should be regarded as targeting—and hence, valid against—*H. abietinum*, *H. parviporum*, *H. irregulare* and *H. annosum s.s*., respectively. The above hosts grew in sites located either in the Alps (*A. alba*, *P. abies*, and *P. sylvestris*) or in the Mediterranean area (*P. pinea*). It is worth noting that most of the Italian forest stands harboring the tree species listed above are located in such ecoregions [41,42], although the distribution areas of *A. alba, P. abies, P. sylvestris*, and *P. pinea* across Europe overlap a wide range of forest types, habitats and ecosystems, whose environmental conditions (e.g., climate, soils, geographic position) and management practices may be highly variable [55,56,57,58]. Although the study sites we selected may be considered representative of the ecological and silvicultural conditions of two distinct and very different ecoregions, the Alpine and the Mediterranean, further studies replicating the same experiments in other ecoregions and areas of Europe (e.g., northern and eastern Europe) are needed to corroborate our core results, which are detailed below.

Overall, considering the results both in terms of the reduction in incidence of the pathogen and of the reduction in area colonized by the pathogen compared to controls, urea was the most effective treatment. This chemical treatment ranked either alone (on *A. alba* and *P. pinea*), or with other treatments (on *P. abies* and *P. sylvestris*) at the lower bound of incidence of *Heterobasidion* spp. Urea performed better than the other treatments in terms of areas colonized by the pathogen, although significance was observed only in the case of *A. alba*. The good performance of urea corroborated previous results obtained using wood discs of the same tree species in controlled conditions [33]. Urea has already been tested against *Heterobasidion* spp. with good results on stumps of *A. alba*, *P. abies* and *P. sylvestris* [10,16,39], and also against *H. annosum s.s.* on stumps of *P. pinea*; however, this is the first report of the efficacy of urea against *H. irregulare* on stumps of such host species. 

Rotstop^®^ was also shown to be rather effective against *Heterobasidion* spp. on stumps of several coniferous tree species, as expected based on a large body of literature and of its widespread use in practical forestry. However, our results should not be regarded as merely confirmatory. In fact, for the first time, this paper provides evidence about the efficacy of Rotstop^®^ on *A. alba* stumps. Furthermore, whereas previous experiments conducted with Rotstop^®^ on *P. pinea* stumps targeted *H. annosum s.s.* [20], this is first report of the efficacy of this treatment against *H. irregulare* on the same host. Adding some additional pieces of evidence on their efficacy, the results obtained with the commercial treatments against *Heterobasidion* spp. urea and Rotstop^®^ could serve as references to appraise the performance of the newly tested treatments based on *P. protegens* strain DSMZ 13134 (i.e., Proradix^®^ and CFF), and on *P. gigantea* MUT 6212 isolated from *P. pinea* in the *H. irregulare* invasion area. With the exception of *P. pinea*, the tested strain of *P. gigantea* performed as well as Rotstop^®^ and, at least on some tree species, as well as urea. Surprisingly, results of *P. gigantea* MUT 6212 on *P. pinea* stumps, which we expected to be well adapted to the host in the Mediterranean region, were undistinguishable from those of untreated control stumps. The reason for this weak efficacy is unknown. Notable is the performance of this treatment especially on *P. sylvestris*. Although the efficacy of Rotstop^®^ on *P. sylvestris* is in agreement with previous research conducted on the same host tree species in Latvia by Kenigsvalde et al. [23], treatment of *P. sylvestris* stumps with the *P. gigantea* MUT 6212 isolated from *P. pinea* was fully effective in preventing pathogen infections. Therefore, we can only speculate that either *P. gigantea* MUT 6212 is better adapted to *P. sylvestris* than to *P. pinea*, or it performs better when inoculated in summer and under Alpine environmental conditions. Although the two hypotheses are clearly not mutually exclusive, this study was designed to compare the performances of treatments on the same tree species and not across species; hence, the above inferences should be regarded as speculation. 

With the exception of applications on *A. alba* stumps, Proradix^®^ failed in reducing the incidence of the pathogen compared to controls, although a significant reduction in the area colonized by the pathogens was observed on the stumps of most tree species. On the other hand, the CFF of *P. protegens* strain DSMZ 13134 performed as well as Rotstop^®^ in reducing the incidence of the pathogen compared to controls, with the only exception being treatments on *P. sylvestris*. CFF of *P. protegens* strain DSMZ 13134 was comparable to the most effective state-of-the-art treatments (urea and Rotstop ^®^) in terms of reducing the areas colonized by the pathogen compared to control stumps, with the exception of *A. alba.* Overall, and with a few exceptions, CFF of *P. protegens* strain DSMZ 13134 performed better than Proradix^®^, supporting previous observations conducted on wood discs under controlled conditions and pointing to a clear role played by antibiosis in the interaction of *P. protegens* strain DSMZ 13134 with *Heterobasidion* spp. [33]. Wood-inhabiting bacteria and fungi may interact in a variety of ways, such as competing for low-molecular-weight compounds released by extracellular fungal enzymes, bacterial mycophagy or the production of toxic bacterial or fungal secondary metabolites [59], but very little is known about the above interactions [60,61]. Oligomers released during lignin degradation by basidiomycetes are appropriate substrates for most wood-inhabiting bacteria that can take advantage of the degradation activity of fungi [59]. In this way, fungi can be systematically deprived of a large part of their growth substrates [59]. By examining the results obtained on *P. abies*, *P. pinea* and *P. sylvestris* stumps, Proradix^®^ was often able to reduce the spreading of the pathogen within stumps (i.e., area colonized by the mycelium), but not its occurrence on stumps (i.e., incidence). Therefore, *Heterobasidion* spp. might colonize wood more rapidly in comparison to *P. protegens* strain DSMZ 13134, by infecting stumps and starting its wood decay process, while the bacterium remains latently present. It should be noted that *P. protegens* strain DSMZ 13134 is a plant-growth-promoting rhizobacteria (PGPR) [62], and may be scarcely adapted to wood. Later, *P. protegens* strain DSMZ 13134 may release secondary metabolites in response to stress signals, thereby affecting *Heterobasidion* spp. Whether or not this scenario is realistic requires further investigations on the patterns of wood colonization by *P. protegens* strain DSMZ 13134 and on its interaction with *Heterobasidion* spp. Conversely, metabolites contained in the CFF of *P. protegens* strain DSMZ 13134 make this treatment ready to go against *Heterobasidion* spp. However, further research aimed at identifying the most active metabolites present in CFF is desirable, because it could lead to significant improvements in the performance of this biological treatment.

This paper is the first focusing on the effectiveness of biological and chemical stump treatments against the invasive *H. irregulare* on *P. pinea*, its main known host in Europe. In the framework of the recently released national regulatory control system for *H. irregulare* [63], data presented in this paper could guide National Plant Protection Organisations in the choice of the most appropriate product for containment. Urea, Rotstop^®^, as well as CFF of *P. protegens* strain DSMZ 13134 are appropriate for this purpose, and hence can be used interchangeably, pending regulatory approvals. Surprisingly, CFF of *P. protegens* strain DSMZ 13134 performed as well as state-of-the-art treatments when applied on stumps of *P. pinea*, although this was not true when treatments were simulated on wood discs of *P. pinea* in controlled conditions [33]. Physical conditions differentiating wood discs and stumps, especially in terms of moisture, may have accounted for this. This finding confirms the need for field tests for the screening of biological control agents, as previously suggested [24].

Experiments carried out on *P. pinea* logs resulted in the good performance of treatments based on *P. gigantea* and on the CFF of *P. protegens* strain DSMZ 13134, and in a lower efficacy of urea and Proradix^®^. A relatively low efficacy of urea on logs could be explained by the mechanisms of action of urea against *Heterobasidion* spp. In fact, the hydrolysis of urea leads to an increase in pH that prevents spore germination on the living tissues of the stump surface [18]; however, such tissues are expected to live shorter in logs than in stumps. Results of the experiment conducted on logs could contribute to the drafting of practical recommendations aimed at preventing infection and the subsequent fruiting of *H. irregulare* on wood residues, should residues be kept on-site for naturalistic purposes. It should be noted that several forests in the outbreak area of *H. irregulare* in Italy are either parks or a Site of Community Importance (SCI) that need to be managed appropriately.

No correlation was observed between stump size and the area colonized by *H. annosum s.l.* in treated stumps of *P. abies*, *P. pinea* and *P. sylvestris*. This finding supports the hypothesis that, generally, the performance of the treatments does not depend upon the stump size. However, a significant correlation between the two variables was found in *A. alba* in both untreated control stumps and stumps treated with Proradix^®^. We cannot exclude that such correlation could have been favored by a longer incubation period of the pathogen on *A. alba* compared to the other tree species (8 months vs. 4 months), making a greater colonization of the fungus on stumps more likely, especially on control stumps and on stumps treated with poorly effective products.

Testing and comparing the efficacy of different stump treatments in the field is pivotal to fine-tune the management of *Heterobasidion* spp. in forest stands. Nonetheless, the availability of chemical or biological products with proven efficacy is not the only prerequisite needed to decide whether stump treatments could be profitably carried out, or not. In fact, a large body of literature suggests that the risk of stump infection should be assessed before applying treatments (see [53,64,65] and the literature therein). Such risks may be highly dependent on climate and on seasonality, influencing the propagule deposition patterns of *Heterobasidion* spp. [9]. Hence, climate change may play an important role in the future by affecting the risk of stump infection by *Heterobasidion* spp. [66,67]. However, our experimental design was not conceived to implement decision-making processes about the opportunity of conducting stump treatments based on the risk of infection. 

In conclusion, this paper provides new evidence supporting the good performances of Rotstop^®^, and especially that of urea, against the three native *Heterobasidion* species on stumps of their preferential hosts and, for the first time, against the North American *H. irregulare* on stumps of *P. pinea*, which is currently a key host of this invasive pathogen in Europe. Although urea may not be longer available as a pesticide against *H. annosum s.l.* and Rotstop^®^ is not approved for use in southern Europe, the CFF of *P. protegens* strain DSMZ 13134 appears a valid alternative to these two state-of-the-art treatments. This is relevant because this treatment, differently from the others, is based on antibiotics contained in a crude cultural cell broth, and could be further improved in terms of efficacy by identifying the active molecules and/or by optimizing their production or application, as previously suggested [33]. In more general terms, the possibility of using different treatments with comparable efficacy may lead to a higher acceptance by citizens of phytosanitary treatments in forests and could also minimize the ecological impact that a large-scale application of a single stump treatment may have.

## 4. Materials and Methods

### 4.1. Study Sites and Treatments 

Stump treatment experiments were conducted in three forest stands in the north-west of Italy dominated by *A. alba*, *P. abies* and *P. sylvestris*, respectively, and in one *P. pinea* stand in central Italy (Table 2). Those forest stands are included in areas known to be infested by *H. annosum s.l.* [3,5]. The study sites in north-west of Italy were typical naturally regenerated, uneven-aged and mixed stands, harboring the relevant tree species in sizeable patches. The study site of central Italy (La Gallinara Park) was an even-aged plantation included in SCIs, preserving coastal Mediterranean habitats. The four study sites were pre-selected based on information on the *Heterobasidion* airspora pointing to the presence, in each stand, of the host-associated *Heterobasidion* species determined in previous studies by using the wood disc exposure method combined with the taxon-specific molecular typing of single spore isolates [4,5,10]. In each site, freshly cut stumps were created during selective thinnings carried out in the frame of the ordinary forest management. Thinnings were conducted on *P. pinea* at La Gallinara Park in January 2020, and on *A. alba*, *P. abies* and *P. sylvestris* in the study sites of north-west of Italy from June to September 2020 (Table 2). Before treatments, all freshly cut stumps were visually inspected for symptoms of wood decay caused by *Heterobasidion* spp., and only asymptomatic stumps were included in the experiments. Thinnings conducted at La Gallinara Park could only include a limited number of trees as prescribed by SCI-related forest regulations, logs deriving from branches of recently felled trees were also used as proxies to simulate *P. pinea* stumps. Logs were approximately 40–50 cm long, with a diameter of 9–33 cm and did not show any visible symptom of decay. Logs were placed upright in the *P. pinea* stand. The diameter of each stump and log was measured along two perpendicular directions on the upper cutting surface.

The efficacy of the following treatments was tested: Proradix^®^ (SP Sourcon Padena GmbH, Tübeningen, Germany), the CFF of *P. protegens* strain DSMZ 13134, a conidial suspension of *P. gigantea* MUT 6212 isolated from fruiting bodies on *P. pinea* at La Gallinara Park (Rome, Italy), the biocontrol product Rotstop^®^ (Verdera Oy, Espoo, Finland), and aqueous urea (Fluka, Cologno Monzese, Italy) solution (30% w/v). *P. protegens* strain DSMZ 13134 was provided by SP Sourcon Padena GmbH (Tübeningen, Germany) and stored in Luria–Bertani (LB) broth amended with 30% glycerol at −80 °C. Fresh cultures were initiated from frozen stocks and refreshed in LB broth at 25 °C for 24 h with shaking before use. CFF preparation was set up based on evidence showing that the antagonistic effect of *P. protegens* (strain DSMZ 13134) against *Heterobasidion* is maximum if CFF is obtained from a pure culture of *P. protegens* (strain DSMZ 13134) at 25 °C [33]. Hence, the CFF was prepared by culturing *P. protegens* (strain DSMZ 13134) in LB broth with constant shaking for 24 h at 25 °C (OD_600_ of 1.1). Cells were pelleted by centrifugation at 4000 rpm for 10 min, and the supernatant was filtered aseptically through a 0.22 μm filter membrane to obtain CFF, free from bacterial cells. The strain of *P. gigantea* MUT 6212 was selected based on its good performances against *Heterobasidion* spp. in in vitro tests [40]. The conidial suspension of *P. gigantea* MUT 6212 was obtained by loading 500 μL of sterile water on the surface of 7-day-old fungal colonies in 9 cm diameter Petri dishes previously incubated at 25 °C in the dark. The water was gently shaken and collected by using a pipette. The concentration of conidia in the suspension was assessed by using a Bürker chamber, and the conidial suspension was subsequently adjusted to 10^4^ conidia mL^−1^. The remaining stump treatments were prepared as described previously [33]. Control stumps received sterile water instead of treatments; we will refer to water as one of the six treatments.

Treatments were carried out manually by spraying the suspensions or solutions onto the surface of freshly cut stumps or logs within 1 hour after their cutting, until the surface became uniformly wet, i.e., an approximately 1 mm thick layer of suspension or solution. At least twenty replicate stumps were used for each treatment in each study site. A total of 135 stumps of *A. alba*, 125 of *P. abies*, 136 of *P. pinea*, and 146 of *P. sylvestris* were included in the experiments. A total of 139 logs of *P. pinea* were used for the experiments, at least 20 for each treatment. For both stumps and logs, treatments were conducted according to a completely randomized design.

### 4.2. Samplings and Laboratory Analyses

Stumps and logs were sampled after 16 weeks from treatments, with the exception of stumps of *A. alba*, which were sampled after 32 weeks due to technical constraints (e.g., a snowy winter). Two 3–5 cm thick half-discs were cut from the top of each stump or log. The upper wood disc was discarded, while the second disc was taken to the laboratory for further analyses; the sampling was performed from one half of the stump or log surface. To avoid the computation of possible infections originating from roots, samples displaying visible symptoms of decay were discarded. The half-discs of stumps or logs were debarked, washed with tap water, and incubated for 10–14 days in plastic bags at room temperature with an optimal relative humidity for fungal growth [10]. Subsequently, the upper surface of the half discs was inspected under a dissecting microscope (20× magnification) for the presence of typical *Heterobasidion* conidiophores, as previously described [4,68]. The area covered with *Heterobasidion* conidiophores was delimited with a marker and measured by using a transparent 1 cm grid. Measurements were expressed as cm^2^ of surface covered with *Heterobasidion* conidiophores.

To determine the species of *Heterobasidion* colonizing the disc surface, diagnostic assays were conducted on five randomly selected discs of control stumps or logs. Isolations were made under a dissecting microscope (20× magnification) with a needle by scraping the surface of conidiophores randomly chosen from the largest infection areas of the control disc. DNA extraction and species typing were conducted as previously described [4,68].

### 4.3. Statistical Analyses

The incidence of *Heterobasidion* spp. for each host species and treatment was calculated in percentage as the ratio between the number of discs colonized by the pathogen and the total number of discs. The exact 95% confidence intervals associated with the incidence values were calculated as reported in Blaker [69]. The effects of the treatments on the incidence of *Heterobasidion* spp. were assessed separately for each host species. In the case of *P. pinea*, the analysis of data was conducted separately for stumps and logs. The treatments were compared by contrasting the incidence of *Heterobasidion* spp. with conditional inference tree models based on unbiased recursive partitioning algorithms [70,71]. The algorithms clustered the treatments based on the following criteria: (1) treatments exerting comparable effects on the incidence of *Heterobasidion* spp. (i.e., treatments resulting in incidence values not significantly different, *p >* 0.05) were grouped within the same cluster; (2) treatments resulting in different effects on the incidence of *Heterobasidion* spp. (i.e., treatments associated with incidence values significantly different, *p <* 0.05) were split in different clusters. Each cluster of treatments was characterized by an overall incidence value of *Heterobasidion* spp., with such values representing a proxy of the expected efficacy of the treatments.

The average area colonized by *Heterobasidion* spp. on the half-surface of stumps and logs was compared among treatments for each host species, separating the analysis of stumps and logs in the case of *P. pinea.* For the above average areas, the 95% bias-corrected and accelerated (BCa) confidence intervals were calculated, as described in DiCiccio and Efron [72]. The above confidence intervals were obtained through the bootstrap iterative resampling method [73], as described in Lione et al. [74]. The comparisons between the average areas colonized by *Heterobasidion* spp. were carried out by running the algorithms fitting the unbiased recursive partitioning conditional inference tree models [70,71]. Algorithms were run on the identity function of the area colonized by *Heterobasidion* spp. as an outcome variable, and on the treatment as an input variable.

The correlation between the area colonized by *Heterobasidion* spp. on the half-surface of the stump and the stump diameter was assessed by testing the significance of the Pearson’s correlation coefficient (R) [75]. The coefficient R was calculated for each treatment and host species.

Statistical analyses were conducted with R version 3.6.0 [76] and with the associated packages bootstrap [77], partykit [70], and binGroup [78]. The significance threshold was set to 0.05 for all tests.

## Figures and Tables

**Table 1:** Pearson’s correlation coefficient between stump size and the area colonized by *Heterobasidion* spp. on stumps of *Abies alba, Picea abies*, *Pinus pinea* and *P. sylvestris* treated with water (wa), Proradix® (pr), cell-free filtrate (cff), *Phlebiopsis gigantea* MUT 6212 (phl), Rotstop® (ro), and urea (ur). The symbol * marks significant correlation (*p* < 0.05).

Host Tree Species	Stump Treatments					
	wa	pr	cff	phl	ro	ur
*A. alba*	0.474 * (*p* = 0.019)	0.447 * (*p =* 0.037)	0.016 (*p* = 0.941)	0.007 (*p* = 0.975)	0.313 (*p* = 0.155)	−0.315 (*p =* 0.163)
*P. abies*	0.220 (*p =* 0.313)	0.250 (*p =* 0.261)	−0.250 (*p =* 0.249)	−0.177 (*p =* 0.406)	−0.065 (*p =* 0.766)	0.017 (*p =* 0.939)
*P. pinea*	0.055 (*p =* 0.794)	−0.036 (*p =* 0.870)	0.004 (*p =* 0.985)	0.047 (*p =* 0.834)	−0.248 (*p =* 0.264)	0.017 (*p =* 0.941)
*P. sylvestris*	−0.230 (*p =* 0.278)	−0.205 (*p =* 0.347)	−0.323 (*p =* 0.122)	NA (*p =* NA)	−0.067 (*p =* 0.757)	−0.167 (*p =* 0.414)

**Table 2:** Main features of the study sites hosting the treatment experiments against *Heterobasidion* spp.

Location	Latitude, Longitude	Elevation (m a.s.l.)	Host Tree Species	Number of Stumps	Stump Diameter, Min–Max (cm)	Mean Stump Diameter ± SD (cm)	Period of Thinning/Treatments	Period of Sampling
La Salle (AO)	45.75667, 7.07907	1001	*A. alba*	135	10–70	24.2 ± 12.7	September 2020	May 2021
Nus (AO)	45.78494, 7.45994	1495	*P. abies*	125	9.5–110	24.7 ± 13.9	June–July 2020	October–November 2020
La Gallinara Park (RM)	41.53156, 12.56187	3	*P. pinea*	136	17–70	39.1 ± 11.0	January 2020	May 2020
Nus (AO)	45.77761, 7.44911	1495	*P. sylvestris*	146	6.5–76	23.9 ± 12.7	June–July 2020	October–November 2020

## Data Availability

All data are reported in the manuscript.
